# Supplementary material for: Complex‐centric proteome profiling by SEC‐SWATH‐MS
Source: Mol Syst Biol. 2019 Jan 14;15(1):e8438. doi: 10.15252/msb.20188438 (PMC6346213; doi:10.15252/msb.20188438)
Supplement: Supplementary file 8 — Dataset EV7 [file MSB-15-e8438-s008.zip › feature_plots_string/Q9ULI4.pdf]

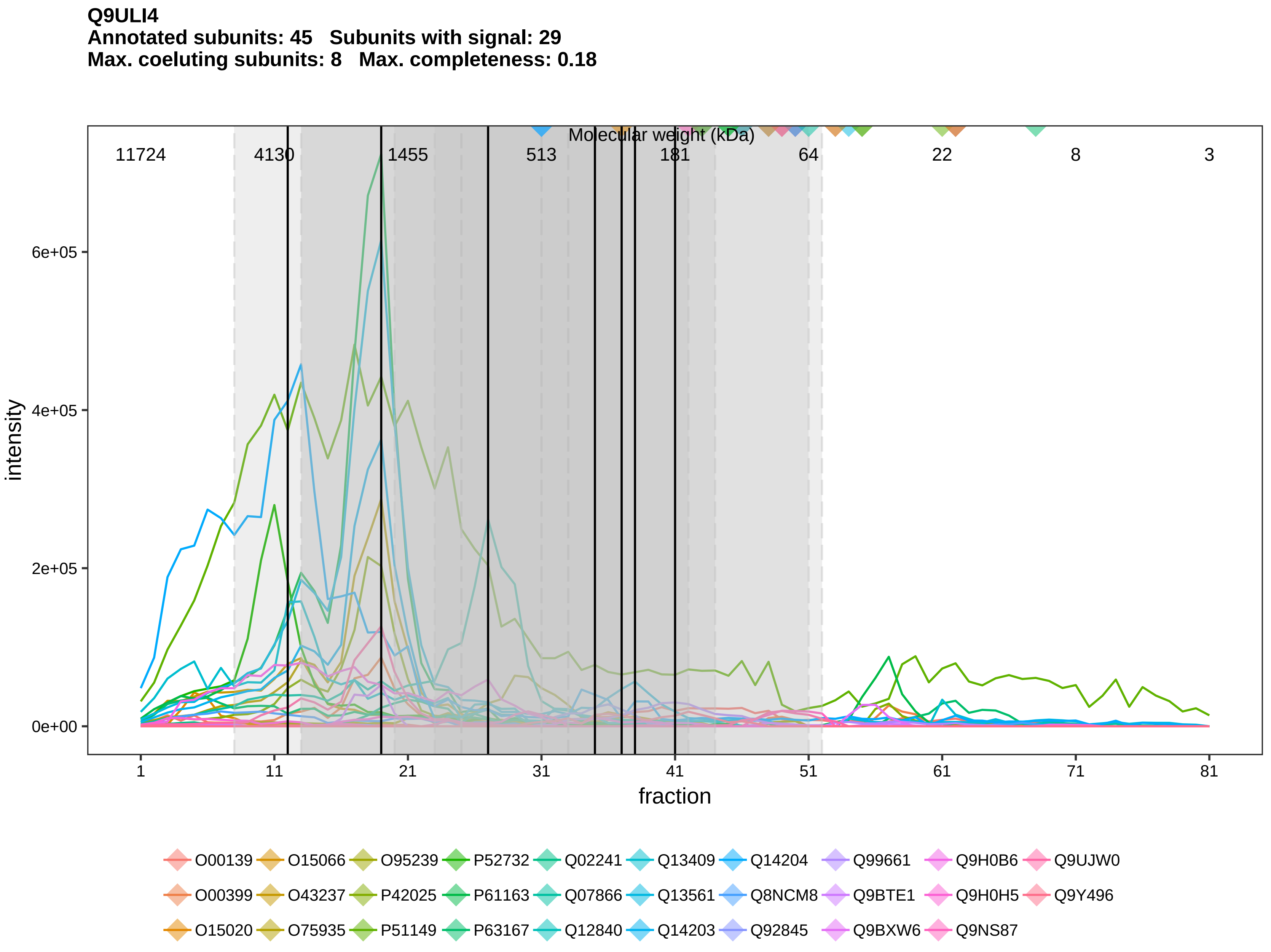
Q9ULI4
Annotated subunits: 45 Subunits with signal: 29
Max. coeluting subunits: 8 Max. completeness: 0.18
Molecular weight (kDa)
11724
4130
1455
513
181
64
22
8
3
intensity
6e+05
4e+05
2e+05
0e+00
1
11
21
31
41
51
61
71
81
fraction
O00139 O15066 O95239 P52732 Q02241 Q13409 Q14204 Q99661 Q9H0B6 Q9UJW0
O00399 O43237 P42025 P61163 Q07866 Q13561 Q8NCM8 Q9BTE1 Q9H0H5 Q9Y496
O15020 O75935 P51149 P63167 Q12840 Q14203 Q92845 Q9BXW6 Q9NS87
